# Supplementary material for: Factors Associated with Vaccination Coverage among 0–59-Month-Old Children: A Multilevel Analysis of the 2020 Somaliland Demographic and Health Survey
Source: Vaccines (Basel). 2024 May 8;12(5):509. doi: 10.3390/vaccines12050509 (PMC11125891; doi:10.3390/vaccines12050509)
Supplement: Supplementary file 1 [file vaccines-12-00509-s001.zip › vaccines-2966831-supplementary.pdf]

Supporting information: Report of vaccination with individual vaccines

Only 108 out of 2673 children had vaccination cards or documents, representing 4% of the total. The information regarding the individual vaccines that the children received was incomplete, with a high percentage of missing data for all individual childhood vaccines. A total of 61.4% of the BCG vaccine, 67.1% of the third dose of the polio vaccine, 71.5% of the third dose of the pentavalent vaccine, and 43.5% of the first dose of the measles vaccine had missing values (S1 Table).

Based on the available cases without missing data (excluding missing values), 35.6%, 28.1%, 48.2%, and 14.2% of those with complete data never received the Bacillus Calmette-Guérin (BCG) vaccine, the third dose of the polio vaccine, the third dose of the pentavalent vaccine and the first dose of the measles vaccine, respectively (S1 Table).

Table S1. The proportion of basic childhood vaccinations among children under five years of age in Somaliland (including missing data) by age in months based on a national expanded program of immunization (EPI) guidelines.

**Having a health card or vaccination document**	**Number**	**Percentage**
**No**	2,565	96%
**Yes**	108	4%
**Total**	2673	100%

**Ever had any vaccinations**	Number	Percentage %	Age group in months	
			Minimum age	Minimum age
**Never vaccinated**	1763	66%	0	56
**Ever vaccinated**	910	34%	0	57
**Total**	2673	100%	..	..

**Bacillus Calmette-Guérin (BCG) vaccine**	Number	Valid data (%)	Percentage %	Age group in months	
				Minimum age	Maximum age
**Not vaccinated**	367	35.6%	13.7%	0	49
**On time vaccinated**	80	7.8%	3%	0	10

**Delayed time vaccinated**	584	56.6%	21.9%	11	56
**Subtotal**	1031	100%	..		
**Missing values**	1642	..	61.4%	0	59
**Grand total**	**2673**	..	100%	---	----
**Polio vaccine dose 3**	Number	Valid data (%)	Percentage %	Age group in months	
				Minimum age	Maximum age
**Not vaccinated**	247	28.1%	9.2%	6	55
**On time vaccinated**	93	10.6%	3.5%	6	18
**Delayed time vaccinated**	169	19.2.0%	6.3%	19	47
**Not applicable**	371	42.1%	13.9%	0	5
**Subtotal**	880	100%	..		
**Missing values**	1793	..	67.1%	6	59
**Grand total**	2673	..	100%	---	----
**Pentavalent vaccine dose 3**	Number	Valid data (%)	Percentage %	Age group in months	
				Minimum age	Maximum age
**Not vaccinated**	368	48.2%	13.8%	3	56
**On time vaccinated**	82	10.7%	3%	3	18
**Delayed time vaccinated**	127	16.6%	4.7%	19	52
**Not applicable**	186	24.4	7%	0	2
**Subtotal**	763	100%			
**Missing values**	1910	..	71.5%	3	59
**Grand total**	2673	..	100%	---	----
**Measles vaccine dose 1**	Number	Valid data (%)	Percentage %	Age group in months	
				Minimum age	Maximum age
**Not vaccinated**	215	14.2%	8%	12	56
**On time vaccinated**[1]	72	4.8%	2.7%	12	15
**Delayed time vaccinated**[2]	537	35.5%	20.1%	16	56
**Not applicable**[3]	687	46.5%	25.7%	0	11
**Sub total**[4]	1511	100%			
**Missing values**	1162		43.5%	12	59
**Grand total**[5]	2673		100%	---	----

[1]Received the vaccine within the time recommended by the WHO

[2] Received the vaccine after the time recommended by the WHO

[3] Received the vaccine before the age scheduled by the WHO

[4] Total valid data without missing values

[5] Total value, including missing values

The prevalence of basic childhood BCG vaccines was 70.4%, the prevalence of polio vaccine dose three was 13.8%, the prevalence of measles vaccine dose one was 63.8%, and the prevalence of pentavalent vaccine dose three was 13.8%.
